# Analysis of cytochrome b_5_ reductase-mediated metabolism in the phytopathogenic fungus *Zymoseptoria tritici* reveals novel functionalities implicated in virulence

**DOI:** 10.1016/j.fgb.2015.05.008

**Published:** 2015-09

**Authors:** Mark C. Derbyshire, Louise Michaelson, Josie Parker, Steven Kelly, Urvashi Thacker, Stephen J. Powers, Andy Bailey, Kim Hammond-Kosack, Mikael Courbot, Jason Rudd

**Affiliations:** a Department of Plant Biology and Crop Science, Rothamsted Research, West Common, Harpenden, Hertfordshire AL5 2JQ, UK; b Department of Biological Chemistry and Crop Protection, Rothamsted Research, West Common, Harpenden, Hertfordshire AL5 2JQ, UK; c Centre for Cytochrome P450 Diversity, Institute of Life Science, College of Medicine, Swansea University Singleton Park, Swansea SA2 8PP, Wales, UK; d Syngenta, Jealott’s Hill, Bracknell, Berkshire RG42 6EY, UK; e Department of Computational and Systems Biology, Rothamsted Research, West Common, Harpenden, Hertfordshire AL5 2JQ, UK; f Bristol University, Senate House, Tyndall Avenue, Bristol BS8 1TH, UK; g Syngenta, Syngenta AG, Schaffhauserstrasse, CH-4332 Stein, Switzerland

**Keywords:** CBR, cytochrome b_5_ reductase, b_5_, cytochrome b_5_, CPR, cytochrome P450 reductase, CYP, cytochrome P450, STB, Septoria tritici blotch, DPI, days post inoculation, WT, wild-type, HPLC, high pressure liquid chromatography, GC–MS, gas chromatography–mass spectrometry, FAME, fatty acid methyl ester, FPKM, fragments per kilobase per million mapped fragments, RNA-seq, RNA sequencing, LCB, sphingolipid long chain base, *Septoria tritici*, *Mycosphaerella graminicola*, Dimorphic fungi, Fatty acids, Cytochrome P450, CYP51

## Abstract

*Septoria tritici* blotch (STB) caused by the Ascomycete fungus *Zymoseptoria tritici* is one of the most economically damaging diseases of wheat worldwide. *Z. tritici* is currently a major target for agricultural fungicides, especially in temperate regions where it is most prevalent. Many fungicides target electron transfer enzymes because these are often important for cell function. Therefore characterisation of genes encoding such enzymes may be important for the development of novel disease intervention strategies. Microsomal cytochrome b_5_ reductases (CBRs) are an important family of electron transfer proteins which in eukaryotes are involved in the biosynthesis of fatty acids and complex lipids including sphingolipids and sterols. Unlike the model yeast *Saccharomyces cerevisiae* which possesses only one microsomal *CBR*, the fully sequenced genome of *Z. tritici* bears three possible microsomal *CBR*s. RNA sequencing analysis revealed that *ZtCBR1* is the most highly expressed of these genes under all *in vitro* and *in planta* conditions tested, therefore Δ*ZtCBR1* mutant strains were generated through targeted gene disruption. These strains exhibited delayed disease symptoms on wheat leaves and severely limited asexual sporulation. Δ*ZtCBR1* strains also exhibited aberrant spore morphology and hyphal growth *in vitro*. These defects coincided with alterations in fatty acid, sphingolipid and sterol biosynthesis observed through GC–MS and HPLC analyses. Data is presented which suggests that *Z. tritici* may use ZtCBR1 as an additional electron donor for key steps in ergosterol biosynthesis, one of which is targeted by azole fungicides. Our study reports the first functional characterisation of CBR gene family members in a plant pathogenic filamentous fungus. This also represents the first direct observation of CBR functional ablation impacting upon fungal sterol biosynthesis.

## Highlights

• Fungi possess a variable often expanded *cytochrome b_5_ reductase* (*CBR*) gene family.
• In the phytopathogen *Zymoseptoria tritici ZtCBR1* is essential for full virulence.
• Sphingolipid, sterol and fatty acid biosynthesis were altered in Δ*ZtCBR1*.
• First report of a *CBR* impacting directly upon fungal sterol biosynthesis *in situ*.

## 1 Introduction

*Septoria tritici* blotch (STB) caused by the wheat leaf-specific Ascomycete fungus *Zymoseptoria tritici* is one of the most economically damaging diseases of wheat worldwide. The most significant ‘within field’ damage caused by *Z. tritici* is mediated through asexual spores. These spores are rain splash propagated throughout the wheat canopy where they attach to leaf surfaces and then germinate into infectious hyphae which penetrate the plant through stomata. This takes place within 24 h of spores landing on the leaf’s surface and is followed by a symptomless phase of slow intercellular colonisation within the leaf lasting approximately 10 days (Orton et al., 2011). Following this initial symptomless period the fungus elicits a rapid onset of host cell necrosis, which bears hallmarks of plant programmed cell death. At this point *Z. tritici* switches to a necrotrophic mode of feeding and begins accumulating biomass rapidly, generating asexual fruiting bodies (termed pycnidia) within the developing necrotic lesions (Keon et al., 2007). Following infection of a susceptible wheat cultivar by a wild-type (WT) strain the complete disease cycle takes approximately 21 days, culminating in the development of mature pycnidia on infected leaves. The masses of asexual spores produced by the pycnidia (pycnidiospores) are multicellular units most frequently composed of four to six cells (Eyal et al., 1987).

There is currently a major deficit in commercially relevant STB-resistant germplasm. As a result *Z. tritici* is a major target for fungicides, especially in temperate regions where it is most prevalent: approximately 70% of fungicides (equating to a cost of >€400 m) sold in the EU are used to prevent STB (O’Driscoll et al., 2014). Due in part to the intense selective pressure caused by the widespread use of just a handful of antifungal chemistries, fungicide resistance in *Z. tritici* is a major problem (Cools and Fraaije, 2008; Siah et al., 2014; Taher et al., 2014). Many fungicides target enzymes involved in electron transfer systems as such systems are often essential for cell function. Therefore, analysis of genes encoding hitherto uncharacterised electron transfer enzymes in *Z. tritici* may be relevant for future development of novel antifungal chemistries.

Aside from those present on the chloroplastic and mitochondrial membranes, the two major eukaryotic electron transfer systems are the cytochrome P450 reductase (CPR)-dependent and microsomal cytochrome b_5_ reductase (CBR)-dependent pathways. The former provides the electrons necessary for the function of cytochrome P450 (CYP) enzymes, which are involved in various biological processes such as detoxification of xenobiotic compounds and biosynthesis and metabolism of lipids and secondary metabolites (George et al., 1998; Mutch et al., 2007; Lepesheva and Waterman, 2007; Richter et al., 2008). The latter usually involves transfer of electrons through CBR then cytochrome b_5_ (b_5_) to terminal electron acceptor desaturase or hydroxylase enzymes. The major functions of the desaturases and hydroxylases in this system are to catalyse double bond formation between carbon atoms and addition of hydroxyl groups during the biosynthesis of unsaturated fatty acids (UFAs) and the more complex lipids the sphingolipids and sterols (Huang et al., 1999; Knutzon et al., 1998; Michaelson et al., 2013; Grinstead and Gaylor, 1982; Moreno-Perez et al., 2011). In addition to its major role in electron transfer to desaturases and hydroxylases, cytochrome b_5_ is also known to be involved in electron transfer to some CYP enzymes (Henderson et al., 2013; Gan et al., 2009), though the functional relationship between CYPs and the CBR-b_5_ pathway remains largely elusive. In addition to the microsomal CBR-b_5_ system there is also a mitochondrial CBR-b_5_ system (Hahne et al., 1994). Though the function of this system also remains largely elusive, it has been linked with metabolism of xenobiotics and lipid biosynthesis (Nikiforova et al., 2014; Neve et al., 2012; Glory and Thiruvenangdam, 2011).

To date studies on microsomal CBR enzymes have been limited to the model yeast *Saccharomyces cerevisiae*, the industrial arachidonic acid-producing fungus *Mortierella alpina* (Class: Zygomycota), the model white-rot fungus *Phanerochaete chrysosporium* (Order: Basidiomycota), and the filamentous fungus *Mucor racemosus* (Class: Zygomycota). In *S. cerevisiae* it was demonstrated that the sole microsomal CBR present in the fully sequenced genome is able to provide the reducing power necessary for the function of a CYP enzyme (albeit in a reconstituted system), CYP51, needed for biosynthesis of the main fungal sterol, ergosterol, rendering CPR dispensable (Lamb et al., 1999; Sutter and Loper, 1989). Similarly, studies in *P. chrysosporium* showed that the CBR-b_5_ system is able to efficiently provide electrons to the enzyme CYP63A2, a multifunctional CYP (Syed et al., 2011). These findings offered some insight into the potential overlap in function between the CPR and CBR systems in fungi. In *M. alpina* two CBRs were identified and cloned in the late 1990s. The first of these was heterologously expressed in *Aspergillus oryzae* leading to an increase in ferricyanide reduction activity (Sakuradani et al., 1999), though no analyses of the *in situ* function of this enzyme were carried out. The second of these was used in conjunction with the first in a phylogenetic analysis, which demonstrated evolutionary divergence of mammalian CBR enzymes from those of fungi and plants (Certik et al., 1999). In *M. racemosus*, CBR was cloned and expressed in *E. coli* (Mirzaei et al., 2010), though again no analyses of the biological function of the enzyme in the host organism were conducted.

Whilst much research has been carried out on mammalian (Celik et al., 2013; Elahian et al., 2014) and plant CBRs (Wayne et al., 2013; Kumar et al., 2006; Shockey et al., 2005; Bagnaresi et al., 2000), there have been no further investigations into the roles of these enzymes in fungi. In order to address this, we have analysed members of the *CBR* gene family in *Z. tritici* which is a plant pathogen in the Dothideomycete class of fungi. In the last decade the molecular interaction between this fungus and its host has come under greater scrutiny (Gohari et al., 2014; Lee et al., 2014; Suffert et al., 2013; do Amaral et al., 2012; Motteram et al., 2009; Marshall et al., 2011). However, there have been little in the way of investigations into the metabolic processes important for growth and plant pathogenesis. Based upon analysis of the fully sequenced genome of the *Z. tritici* reference isolate IPO323 (Goodwin et al., 2011), and recent RNA sequencing (RNA-seq) data (Rudd et al., 2015) we determined that only one of the three putative *CBR*s in *Z. tritici*, *ZtCBR1*, was highly expressed both *in vitro* and throughout plant infection. By generating targeted *ZtCBR1* disruption strains it was shown that this gene is essential for full virulence in wheat. *In vitro* observation of Δ*ZtCBR1* strains revealed various morphological and biochemical defects including reduced spore size, reduced filamentous growth and almost a complete lack of asexual sporulation at the end of the infection cycle *in planta*. Perturbations in sphingolipid, sterol and fatty acid biosynthesis pathways were identified using GC–MS and HPLC analyses in the Δ*ZtCBR1* strain. This study represents the first functional analysis of members of the *CBR* gene family in a plant pathogenic Ascomycete fungus, and highlights several *CBR1*-regulated functions that underpin virulence in *Z. tritici*.

## 2 Materials and methods

### 2.1 Identification of sequence homologues of yeast cytochrome b_5_ reductase (ScCBR1) in *Z. tritici* and other filamentous fungi

In order to identify putative microsomal *CBR* genes in several unrelated Ascomycete and Basidiomycete fungi including *Z. tritici*, BLASTp analyses were carried out via the NCBI BLASTp suite (http://blast.ncbi.nlm.nih.gov/Blast.cgi?PROGRAM=blastp&PAGE_TYPE=BlastSearch&LINK_LOC=blasthome) using the *S. cerevisiae* CBR sequence, ScCBR1 (GenBank accession: CAA82214.1), as a query (fungal genomes queried are detailed in Supplementary Table 2).

BLAST hits above 30% amino acid (aa) identity with an e value lower than e^−10^ were retrieved and subjected to PFam domain prediction (Finn et al., 2014) to determine whether they contained flavin adenine dinucleotide (FAD)-binding (PFam identifier: PF00667) and nicotinamide adenine dinucleotide (NAD)-binding domains (PFam identifier: PF08030), which are both universally present in CBRs. Sequences that contained additional molybdopterin-binding domains (PFam identifier: PF00174) were excluded from further analyses as this is a feature common to nitrate reductases, which are structurally closely related to CBRs but involved in different processes (Truong et al., 1991).

The putative microsomal CBR sequences were then subjected to a TargetP analysis (Emanuelsson et al., 2000) (accessed via: http://www.cbs.dtu.dk/services/TargetP/) to determine whether the predicted proteins were potentially localised to mitochondria or the endoplasmic reticulum. The *S. cerevisiae* mitochondrial CBR sequence, ScMCR1 (GenBank accession: NP_012221.2), was then used as a query in further BLASTp analyses to determine whether CBRs identified with a TargetP prediction of localisation to mitochondria were indeed more closely related to this sequence than SsCBR1. This step was needed to differentiate between sequence homologues as mitochondrial CBRs are highly similar to microsomal CBR sequences owing to the presence of the highly conserved FAD- and NAD-binding domains necessary for their functions.

### 2.2 *In vitro* culture conditions and fungal strains

Fungal spores were routinely cultured on yeast peptone dextrose (YPD) agar for six days at 18 °C. For RNA-seq analysis spores of the WT reference isolate IPO323 were grown in Czapek Dox broth (CDB) minimal medium and potato dextrose broth (PDB) nutrient-rich medium. Fungal cultures were propagated in shaking flasks at 220 rpm and 18 °C for 3 days for PDB or 5 days for CDB and then harvested via vacuum filtration, as detailed in Rudd et al. (2015). These incubation periods were determined to be within the logarithmic growth phase for *Z. tritici*. For analysis of fatty acid methyl ester (FAME), sphingolipid long chain base (LCB) and sterol content the Δ*Ku70* strain (treated as WT) Bowler et al., 2010 and the Δ*ZtCBR1-1* strain generated in this study were propagated under the same conditions in yeast peptone dextrose (YPD) liquid medium. Spores were harvested via vacuum filtration after 4 days of growth and snap-frozen in liquid nitrogen. Prior to analysis of sterol content, spores were freeze-dried. The same culture conditions were used to grow spores for microscopy. To induce filamentous growth *in vitro*, spores were spot-inoculated onto 1% agar from a spore suspension of 1 × 10^6^ spores ml^−1^ according to (Motteram et al., 2011).

### 2.3 Growth and inoculation of plants

Seventeen day old seedlings of the STB-susceptible wheat cultivar Riband were used for all plant infection assays and RNA-seq analysis. Seeds were pre-germinated on wet sand at 10% relative humidity for 3 days prior to potting and subsequently kept with a 16 h daylight cycle. Adaxial surfaces of second leaves were inoculated according to (Keon et al., 2007) with spore suspensions at a density of 2 × 10^6^ spores ml^−1^ in 0.1% Silwet in sterile water. For infection assays, mock leaves were inoculated with 0.1% Silwet only. Plant inoculation for RNA-seq analysis conducted in the previous study (Rudd et al., 2015) followed the same procedure, without the pre-germination step, using a spore density of 10^6^ spores ml^−1^ in 0.1% Tween20 in sterile water.

For analysis of asexual fungal sporulation, 8–12 replicate leaves from independently inoculated wheat seedlings randomly distributed in a walk-in temperature, humidity and light-controlled artificial environment were collected at either 21 or 34 days post inoculation (DPI). For the previously published RNA-seq data, each of two biological replicate plant samples were made up of 5 leaves collected from independent plants randomly distributed in a single walk-in temperature and humidity-controlled glasshouse. Samples were collected at one, four, nine, 14 and 21 DPI. Leaves collected for RNA-seq were immediately frozen in liquid nitrogen, freeze-dried, then ground to fine powder in liquid nitrogen before RNA extraction.

### 2.4 RNA extraction and RNA sequencing

All aspects relating to RNA-seq analysis of fungal gene expression during growth in Czapek-Dox and Potato Dextrose broths and at five time points of plant infection are described in detail in a previous study (Rudd et al., 2015). To summarise, the following principle procedures were followed: Total RNA was isolated from freeze-dried tissues using the Trizol procedure (Chomczynski and Sacchi, 1987) incorporating a final LiCl_2_ precipitation. All samples (single-end) were mapped with TopHat (v2.0.6) against the *Z. tritici* genome (-G Mycosphaerella_graminicola.MG2.16.gtf) (Trapnell et al., 2012). Cufflinks (v2.1.1) was used to calculate FPKM values for reference annotations (-G Mycosphaerella_graminicola.MG2.16.gtf) but excluding genes annotated with rRNA (-M rRNA_genes.gtf). Differential expression analysis was done with cuffdiff (cuffdiff -u -M rRNA_genes.gtf -b Mycosphaerella_graminicola.MG2.16.dna.toplevel.fa).

### 2.5 *Agrobacterium tumefaciens* – mediated targeted disruption of fungal genes

In order to target *ZtCBR1* for gene function ablation the plasmid pNOV2114 was used (Motteram et al., 2009). Two sequences flanking *ZtCBR1* were obtained from the JGI genome for *Z. tritici* (http://genome.jgi.doe.gov/Mycgr3/Mycgr3.home.html) and PCR amplified. Amplified sequences were then purified and inserted into pNOV2114 either side of a hygromycin resistance cassette (*hph*) (also inserted as purified PCR product) under a *trpC* promoter. The same procedure was used to generate transformation vectors for *ZtCBR2* and *ZtCYP-24*, though the plasmid used was pCHYG (Motteram et al., 2009), which already contained *hph* under the *trpC* promoter. All primers and added restriction sites used for these procedures are detailed in Supplementary Table S1 and Supplementary Fig. S1, A shows diagrams indicating positions and sizes of flanking sequences relative to genes of interest.

*Agrobacterium tumefaciens* – mediated transformation of *Z. tritici* spores was carried out according to (Zwiers and De Waard, 2001) with slight modifications. Instead of using the antibiotic Cefotaxim, Timentin was used in transformant selection plates as it was found to be more efficient for removal of residual *Agrobacterium tumefaciens* after transformation. All transformations were carried out in a Δ*Ku70* background as disruption of this gene has been shown to prevent ectopic insertion whilst maintaining WT growth and virulence (Bowler et al., 2010); the Δ*Ku70* strain used in transformation was treated as WT in all subsequent experiments.

Transformant colonies were sub-cultured twice on hygromycin-selective agar (50 ug ml^−1^). To confirm integration of *hph+trpC* at the desired locus and in the correct orientation, a primer from within *hph* and within 200 bp of a flanking region used to guide insertion were used to amplify a diagnostic region of approximately 1.5 kb. A single band in gDNA of mutant strains and lack thereof in gDNA of the WT was considered representative of successful targeted disruption. All primers used for these procedures are detailed in Supplementary Table 1. At least three disruption strains were generated for each gene of interest (Supplementary Fig. S1, B). Two Δ*ZtCBR1* strains were carried forward for further analysis and arbitrarily named Δ*ZtCBR1-1* and Δ*ZtCBR1-2*. Only single Δ*ZtCBR2* and Δ*ZtCYP-24* strains were fully analysed in planta as preliminary testing of several strains showed no apparent reductions in virulence.

### 2.6 Quantification of asexual sporulation in infected leaf tissue

To assess the degree of asexual sporulation exhibited by *Z. tritici* strains at the stated time points post inoculation, leaf samples were kept for a further 48 h at 100% relative humidity in darkness at 18 °C. Leaves were then submerged in 1 ml of distilled water and left overnight. Submerged leaves were vortexed for ∼10 s and spores released into the water were counted using a haemocytometer. A total of 8–12 leaves each taken from individual wheat seedlings were analysed this way for each fungal strain tested. Analysis of variance (ANOVA) was used on raw data for Δ*ZtCYP-24* and Δ*ZtCBR2* and log_e_^2^-transformed data for the Δ*ZtCBR1* strains alongside the WT to assess differences in the amount of asexual sporulation. When a significant difference (*p* < 0.05, *F*-test) was found, Fisher’s least significant difference (LSD) test was used to determine significant (*p* < 0.05) pairwise differences between strains.

Micrographs of mutant and WT spores were generated via bright field imaging using a Zeiss LSM 780 microscope (Carl Zeiss AG, Oberkochen, Germany). In order to visualise cell boundaries within spores, spore-suspensions (10^6^ spores ml^−1^ in sterile distilled water) were stained for five minutes at room temperature with the fluorescent cell wall stain calcofluor white at a 1% concentration and imaged using the same microscope with a 405 nm laser. Distance between septa and number of individual cells in each spore were then assessed using ImageJ software (Schneider et al., 2012). Spores from three replicate cultures were included in these analyses and for each replicate culture a minimum of 11 spores were assessed. Differences in mean cell length and number of spores between strains were assessed using ANOVA on log_e_^2^-transformed data treating each individual spore as a technical replicate and each culture as a biological replicate. This was followed by Fisher’s LSD test to determine significant pairwise differences between strains. To determine differences in mean proportion of spores with only one cell present between strains, two sample binomial tests were used to compare the WT with each individual strain, Δ*ZtCBR1*-*1* and Δ*ZtCBR1*-*2*. Statistical analyses were carried out using the GenStat (2014, 17th edition, © VSN international Ltd, Hemel Hempstead, UK) statistics package.

### 2.7 GC–MS analysis of sterol content of Δ*ZtCBR1*-*1*

Samples from four independent liquid cultures were analysed. Each sample of 20 mg fresh weight (freeze-dried) was used for replicate analyses and results from WT and the Δ*ZtCBR1*-*1* mutant strain were compared using Student’s *t*-tests. Non-saponifiable lipids were extracted as reported previously (Kelly et al., 1995). Samples were dried in a vacuum centrifuge (Heto) and derivatized by addition of 100 μl of 90% bis(trimethylsilyl)-trifluoroacetamide (BSTFA) – 10% trimethylsilyl (TMS) (Sigma–Aldrich) and 200 μl anhydrous pyridine (Sigma–Aldrich) and heating for 2 h at 80 °C. Gas chromatography–mass spectrometry was performed using a VG12-250 mass spectrometer (VG Biotech) with splitless injection. Individual sterols were identified by reference to relative retention times, mass ions, and fragmentation patterns. Data were analysed using MSD Enhanced ChemStation (Agilent Technologies). Statistical analyses were carried out using the GenStat (2014, 17th edition, 297 © VSN international Ltd, Hemel Hempstead, UK) statistics package.

### 2.8 GC–MS analysis of fatty acid methyl ester content of Δ*ZtCBR1*-*1*

Samples from five independent liquid cultures were analysed. Each sample of 20 mg fresh weight was used for replicate analyses, and results from WT and the Δ*ZtCBR1*-*1* mutant strain were compared using Student’s *t*-tests. Lipids were extracted and methylated as described (Garces and Mancha, 1993) with minor modifications. Methyl heptadecanoate (C17:0) was added to samples as an internal standard. Following methylation the heptane fraction was concentrated and re-suspended in 300 μl solvent prior to injection of 1 μl onto the GC column. Methyl ester derivatives of total fatty acids extracted were analysed by GC (Agilent 7890A) using an Agilent DB-225 column (30 m × 0.32 mm × 0.3 μm). Inlet and detector temperature was set to 250 °C and 1 μl of each sample was analysed using splitless injection and a constant flow rate of 2 ml min^−1^. The oven temperature cycle was set as follows: a start temperature of 50 °C was held for 1 min to allow vaporised samples and the solvent (hexane) to condensate at the front of the column. Oven temperature was then increased rapidly to 190 °C at a rate of 40 °C min^−1^ followed by a slower increase to 220 °C at a rate of 1.5 °C min^−1^. The final temperature of 220 °C was held for 1 min giving a total run time of 25 min 50 s per sample. Fatty acid methyl esters (FAMEs) were detected using a Flame Ionisation Detector (FID). Chromatograms were analysed using the offline session of the Agilent ChemStation software (Agilent Technologies). The retention time and identity of each fatty acid methyl ester (FAME) peak was calibrated using the FAME Mix Rapeseed oil standard (Supelco). Statistical analyses were carried out using the GenStat (2014, 17th edition, © VSN international Ltd, Hemel Hempstead, UK) statistics package.

### 2.9 HPLC analysis of sphingolipid long chain 320 base content of Δ*ZtCBR1*-*1*

Samples from four independent liquid cultures were analysed. Each sample of 20 mg fresh weight was used for replicate analyses and results from WT and the Δ*ZtCBR1*-*1* mutant strain were compared using a Student’s *t*-test. A 2 μg aliquot of d20:0 LCB was used as the internal standard. Long chain bases (LCBs) were liberated from material by an alkaline hydrolysis extraction method based on (Sperling et al., 1998). Briefly, this was performed using 10% BaOH and dioxane 1:1 v/v in capped tubes overnight at 110 °C. These were then cooled and extracted with chloroform/dioxane/water (8/3/8, v/v/v). The LCB fraction was converted to dinitrophenyl derivatives with 0.2 ml 0.5% (v/v) methanolic 1-fluoro-2,4-dinitrobenzene and 0.8 ml 2 M boric acid/KOH at 60 °C for 30 min. LCBs were then extracted by phase partitioning with CHCl_3_/methanol/H_2_O, 2:1:1 (v/v/v). The organic phase was removed and washed with an equal volume of 0.1 M KOH and 0.5 M KCl. The organic phase was then blown down and resuspended in 200 μl MeOH for analysis. Analysis by reverse-phase HPLC was performed using a C18 RP 250 × 4 mm column with a flow rate of 1 ml min^−1^ and a concave gradient from 80% to 100% methanol/acetonitrile/2-propanol, 10:3:1 (v/v/v), against water in 45 min. The elution was monitored with ESI-MS/MS MRM on a 4000 QTRAP and at a wavelength of 350 nm on an Agilent 1200 HPLC. Statistical analyses were carried out using the GenStat (2014, 17th edition, © VSN international Ltd, Hemel Hempstead, UK) statistics package.

## 3 Results

### 3.1 The *Z. tritici* genome encodes three putative microsomal CBRs, alike many other genomes of filamentous fungi

**Fig. 1:**
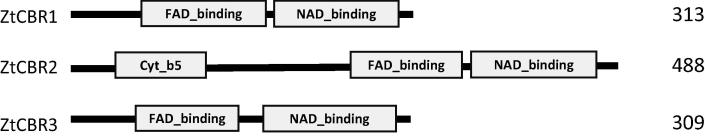
Structural characteristics of the three putative *Z. tritici* microsomal CBR proteins. PFam domains of the three *Z. tritici* putative microsomal CBR sequences retrieved. Each predicted protein sequence contains FAD-binding (PFam identifier: PF00667) and NAD-binding (PFam identifier: PF08030) domains canonical for CBR sequences. ZtCBR2 also contains a b5-fusion domain (PFam identifier: PF00173) at the N-terminus. Amino acid sequence length is given to the right.

In order to investigate the presence of putative microsomal CBR sequences in 21 fungal genomes derived from pathogens and non-pathogens, in both the Basidiomycete and Ascomycete phyla, BLASTp analyses were conducted using the *S. cerevisiae* CBR sequence ScCBR1 as a query. Sequences retrieved were subjected to a TargetP analysis to determine whether the predicted proteins were likely to be localised to mitochondria or microsomes. This analysis identified three putative microsomal CBR proteins in *Z. tritici*. The putative microsomal CBR sequences were named ZtCBR1-3 in order of similarity to SsCBR1 (GenBank accessions: XP_003854385.1, XP_003852872.1 and XP_003847738.1; and Ensembl identifiers: Mycgr3T69942, Mycgr3T57682 and Mycgr3T51378, respectively for ZtCBR1, ZtCBR2 and ZtCBR3). These three sequences contained both NAD- and FAD-binding domains canonical for CBRs (Fig. 1); the ZtCBR2 sequence also contained a cytochrome b_5_ fusion domain (PFam identifier: PF00173) at the N-terminus (Table 1 and Fig. 1).

This higher number of microsomal CBR sequences relative to *S. cerevisiae* was also seen amongst distantly related fungi. For example the non-pathogenic fungus *Aspergillus nidulans* contained seven putative microsomal CBRs, the highest number observed for the fungal species analysed in this study. The lowest number of putative microsomal CBRs found was in *Mycosphaerella musiva*, a plant pathogenic Dothideomycete fungus related to *Z. tritici* which contained only a single CBR sequence lacking a TargetP-predicted mitochondrial localisation. All sequences identified that were predicted to be localised to mitochondria through TargetP analyses showed higher similarity to SsMCR1 than SsCBR1, which was initially used to retrieve them. In the species *A. terreus*, *Colletotrichum graminicola*, *Dothistroma septosporum* and *Mycosphaerella fijiensis*, two to three CBR sequences with TargetP-predicted mitochondrial localisation and higher similarity to SsMCR1 were retrieved using SsCBR1 as a query sequence. In addition to NAD and FAD-binding domains, PFam analyses also identified b_5_ fusion domains in a number of putative microsomal CBR sequences other than ZtCBR2; these were all present at the N-terminus.

No obvious link between number of CBR sequences and fungal lifestyle was apparent except for the lack of CBR sequences observed in four of the five endophytic fungal species analysed (Table 1). The data suggest that many filamentous fungi (perhaps excluding certain endophytic species) have a greater diversity of CBRs relative to the Ascomycete yeast *S. cerevisiae*.

### 3.2 RNA sequencing analysis demonstrates high constitutive expression of *ZtCBR1* and lower expression with transient up-regulation of *ZtCBR2 in planta*

**Fig. 2:**
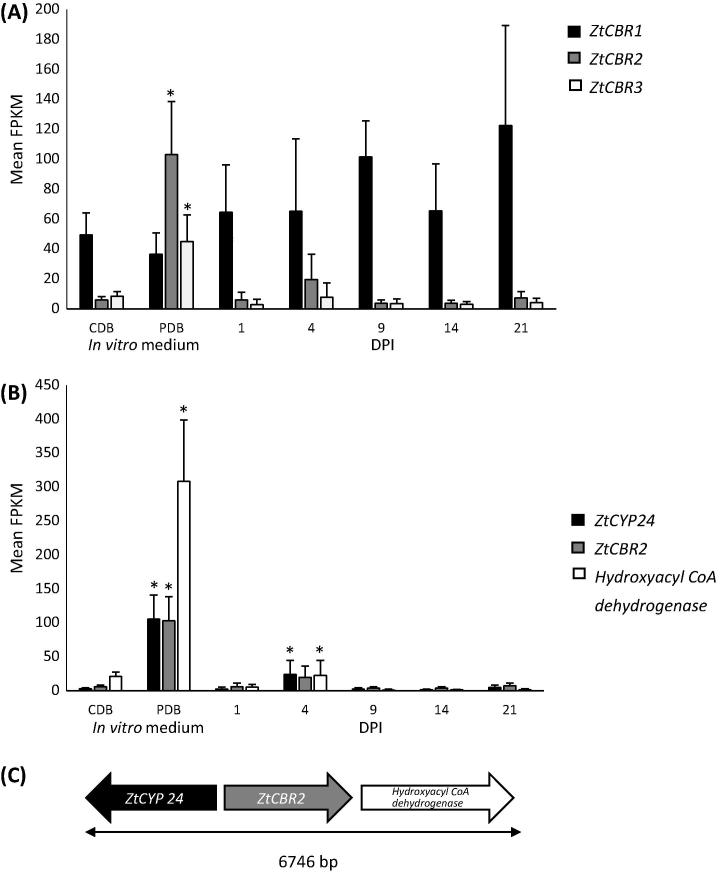
Expression profiles of *CBR* and related genes in *Z. tritici*. (A) Mean FPKM values showing expression of *Z. tritici CBR*s in Czapek Dox broth (CDB), potato dextrose broth (PDB) and at one, four, nine, 14 and 21 days post inoculation (DPI) of wheat leaves; significant differences in expression of *ZtCBR2* and *ZtCBR3* (^*^ *p* < 0.05) were found during growth in PDB relative to during growth in CDB. (B) Mean FPKM values showing expression profile of *ZtCBR2* and the two neighbouring genes, *ZtCYP-24* and a putative *hydroxyacyl CoA dehydrogenase* across the same set of conditions. (C) A diagram showing gene organisation across the region with the total size of the putative three gene cluster in base pairs indicated below. The two genes neighbouring *ZtCBR2* were significantly up-regulated both in PDB and on day four of infection (^*^ *p* < 0.05) relative to during growth in CDB; *ZtCBR2* exhibited a similar expression profile though apparent up-regulation was only significant in PDB (*p* < 0.05).

In order to identify genes that might be important for infection, an RNA-seq analysis was conducted in a previous study (for details see Rudd et al., 2015) on samples taken from two *in vitro* growth conditions and five *in planta* infection time points including one, four, nine, 14 and 21 days post inoculation (DPI). These time points are representative of major transitions in fungal growth; day one and day four representing the symptomless phase, day nine representing the transition to necrosis, day 14 necrotrophic growth and day 21 asexual sporulation. Analysis of mean FPKM values showed that *ZtCBR1* was highly expressed in both *in vitro* conditions and at all tested infection time points (Fig. 2A). *ZtCBR2*, though generally less expressed overall, was up-regulated in the nutrient-rich medium PDB (*p* < 0.05) relative to during growth in CDB, and again (although data were not significant) on day four of infection relative to during growth in CDB. In addition, two genes neighbouring *ZtCBR2* in the *Z. tritici* genome, annotated as a predicted *CYP*, *ZtCYP-24* (GenBank accession: XP_003853538.1), and a predicted *hydroxyacyl coA dehydrogenase* (GenBank accession: XP_003852872.1), showed similar expression profiles to *ZtCBR2*. Both genes exhibited a significant up-regulation in PDB (*p* < 0.05) and up-regulation on day four of infection (*p* < 0.05) either relative to during growth in CDB for *ZtCYP-24* or to all other infection time points for the *hydroxyacyl CoA dehydrogenase* (Fig. 2B). *ZtCBR3* displayed a similar (albeit lower level) expression pattern to *ZtCBR2* and was significantly up-regulated during growth in PDB relative to during growth in CDB (*p* < 0.05) (Fig. 2A).

### 3.3 Fungal gene deletion and wheat leaf infection assays demonstrate an important role for Δ*ZtCBR1* in disease progression and asexual sporulation in *Z. tritici*

Based on the previous gene expression analysis, and in order to assess the roles for different *CBR*s and *CYP*s in fungal growth and virulence, various gene disruption strains were generated in the Δ*Ku70* strain of *Z. tritici* IPO323 and PCR-verified (Supplementary Fig. 1). These were then tested for the ability to cause disease on the STB-susceptible wheat cultivar Riband (Keon et al., 2007).

**Fig. 3:**
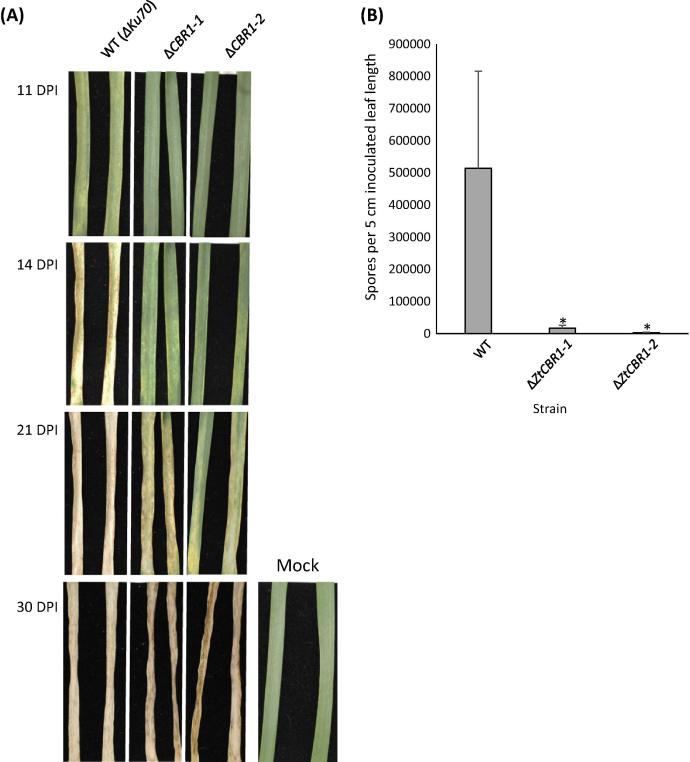
Δ*ZtCBR1* mutants show delayed disease symptom induction and strongly reduced asexual sporulation on wheat leaves. (A) Leaves infected with WT, Δ*CBR1*-*1* and Δ*ZtCBR1*-*2* after 11, 14, 21 and 30 DPI; mock-inoculated control leaves at 30 DPI are shown to the right. A total of eight leaves per strain/mock were inoculated and two representative leaves are shown. (B) Mean number of spores (recovered by washing) per 5 cm length of inoculated leaf for WT, Δ*ZtCBR1*-*1* and Δ*ZtCBR1*-*2* after 34 DPI showing a significant reduction in asexual sporulation of the mutant strains relative to the WT (^*^ *p* < 0.05).

Two independent CBR1 mutants, Δ*ZtCBR1*-*1* and Δ*ZtCBR1*-*2*, both caused delayed symptom manifestation *in planta*. At 11 DPI when symptoms first appeared in the WT, neither Δ*ZtCBR1*-*1* nor Δ*ZtCBR1*-*2* had caused any symptoms. At 14 DPI when the WT had caused substantial host necrosis, only limited chlorosis was apparent in leaves infected with Δ*ZtCBR1*-*1* and Δ*ZtCBR1*-*2*. After 21 days, leaf necrosis and pycnidiation were apparent in the WT but only patchy chlorosis was apparent in Δ*ZtCBR1*-*1* and Δ*ZtCBR1*-*2*. After 30 days, necrosis was observed in leaves inoculated with Δ*ZtCBR1*-*1* and Δ*ZtCBR1*-*2*, though pycnidia were not visible (Fig. 3A). In contrast to wild-type infections, no pycnidia were visualised on Δ*ZtCBR1-*infected leaves even when assays were allowed to proceed for a further 14 days (at 44 DPI – data not shown). Quantitative analysis of asexual sporulation performed at 34 DPI, demonstrated that both Δ*ZtCBR1*-*1* and Δ*ZtCBR1*-*2* produced significantly reduced asexual spore-numbers (*p* < 0.05) relative to the WT (Fig. 3B).

In contrast all other mutant strains except for Δ*ZtCBR1*-*1* and Δ*ZtCBR1*-*2* caused WT symptoms *in planta* with no changes in asexual spore counts (Supplementary Fig. S2). After 14 days cell death was apparent in leaves inoculated with WT, Δ*ZtCYP-24* and Δ*ZtCBR2* strains. After 21 days symptoms had progressed to widespread necrosis at the site of inoculation, and necrotic lesions contained numerous pycnidia. For all strains a total of 12 leaves were evaluated, each showing symptoms consistent with the next. Two representative leaves are shown for each assay in Supplementary Fig. S2, A. Asexual sporulation did not appear to be affected by functional ablation of *ZtCYP-24* and *ZtCBR2*, as evidenced by retrieval of a WT amount of asexual spores from infected leaves after 21 days (Supplementary Fig. 2B).

### 3.4 Δ*ZtCBR1* spores have altered morphology and transition more slowly to filamentous growth

**Fig. 4:**
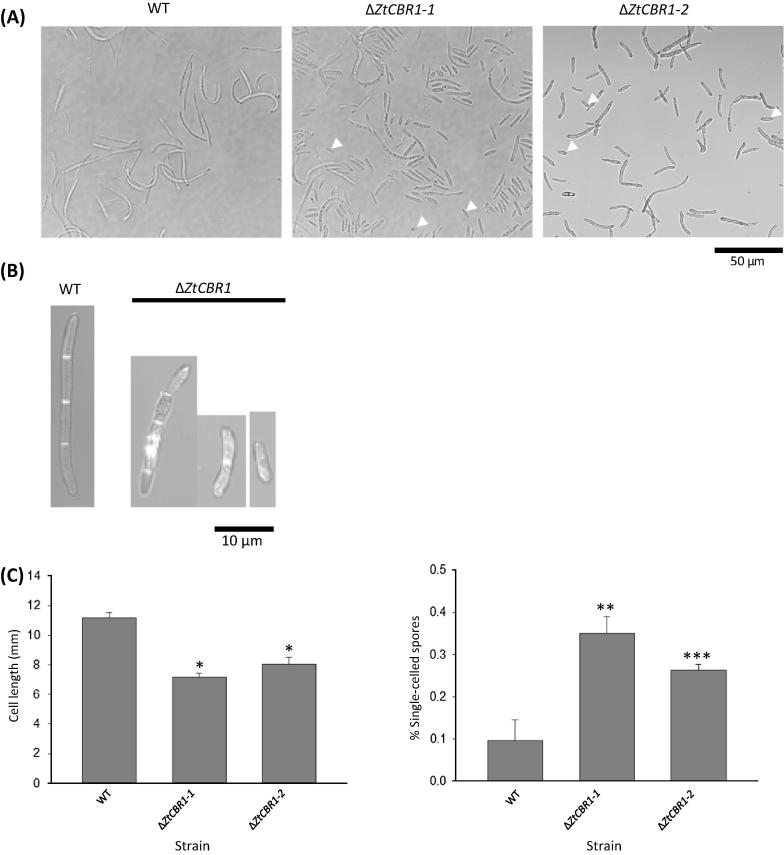
Δ*ZtCBR1* mutants show abnormal spore morphologies. (A) Bright field imaging of WT and Δ*ZtCBR1* spores. Arrowheads highlight examples of single-celled spores more frequently observed in Δ*ZtCBR1.* (B) Representative spores of WT and Δ*ZtCBR1* strains stained with calcofluor white. (C) Mean cell length for WT and two Δ*ZtCBR1* strains. Bars represent standard error (^*^ *p* < 0.05). (D) Mean percentage of single-celled spores for WT and two Δ*ZtCBR1* strains. Bars represent standard error (^**^ *p* < 0.01, ^***^ *p* < 0.001).

In order to capture morphological defects observed from bright field and laser scanning microscopic imaging of Δ*ZtCBR1* strains (Fig. 4A and B), both individual cell length (defined as distance between septa in the multicellular spores) and the number of cells per spore were assessed. These microscopic analyses revealed that both Δ*ZtCBR1* strains exhibited a significant overall decrease in individual cell length (mean length, WT = 11.14 μm, Δ*ZtCBR1*-*1* = 7.17 μm, Δ*ZtCBR1*-*2* = 7.97 μm, *p* < 0.05) and increase in the proportion of single-celled spores (binomial test Δ*ZtCBR1*-*1* vs WT, *p* = 0.003, Δ*ZtCBR1*-*2* vs WT *p* < 0.001) (Fig. 4C and D).

**Fig. 5:**
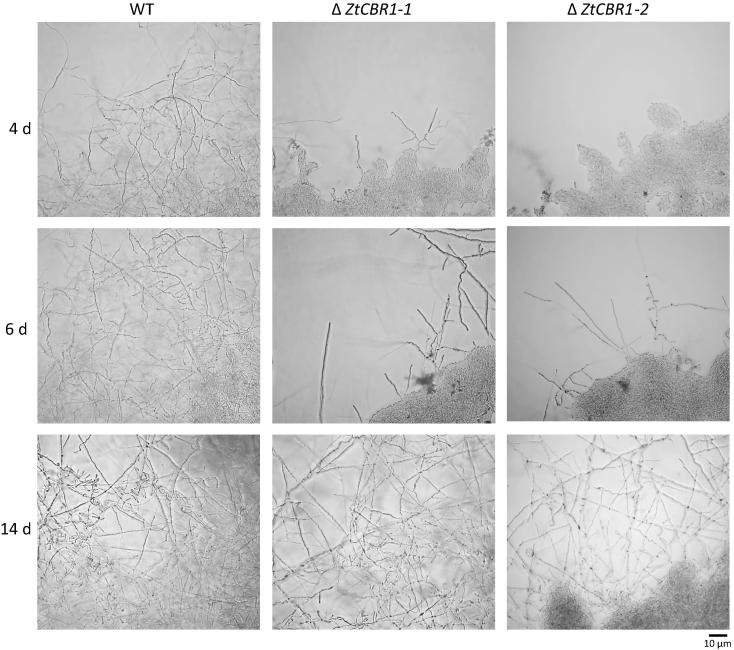
Δ*ZtCBR1* mutants show reduced frequency and rate of hyphal growth. Micrograph showing the appearance of radial hyphal growth produced from the edge of a 5 μl spore droplet for WT and the two Δ*ZtCBR1* strains after four, six and fourteen days (d) of growth on 1% water agar.

In order to infect wheat leaves, *Z. tritici* spores require differentiation into hyphae. It is thought that hyphal growth is induced when *Z. tritici* is exposed to low nutrient environments such as on the leaf surface, or in sterilised water culture and water agar *in vitro*. In order to assess defects in hyphal growth in both Δ*ZtCBR1* strains, spores were spot inoculated onto 1% water agar plates and grown for two weeks. After four days WT spores had formed an extensive hyphal network, whereas Δ*ZtCBR1* spores had produced no hyphae. However, after six days limited hyphal growth was observed for both Δ*ZtCBR1* strains, and after two weeks the mycelium exhibited almost WT filamentous growth (Fig. 5).

### 3.5 Δ*ZtCBR1*-*1* exhibits an altered fatty acid methyl ester profile

**Fig. 6:**
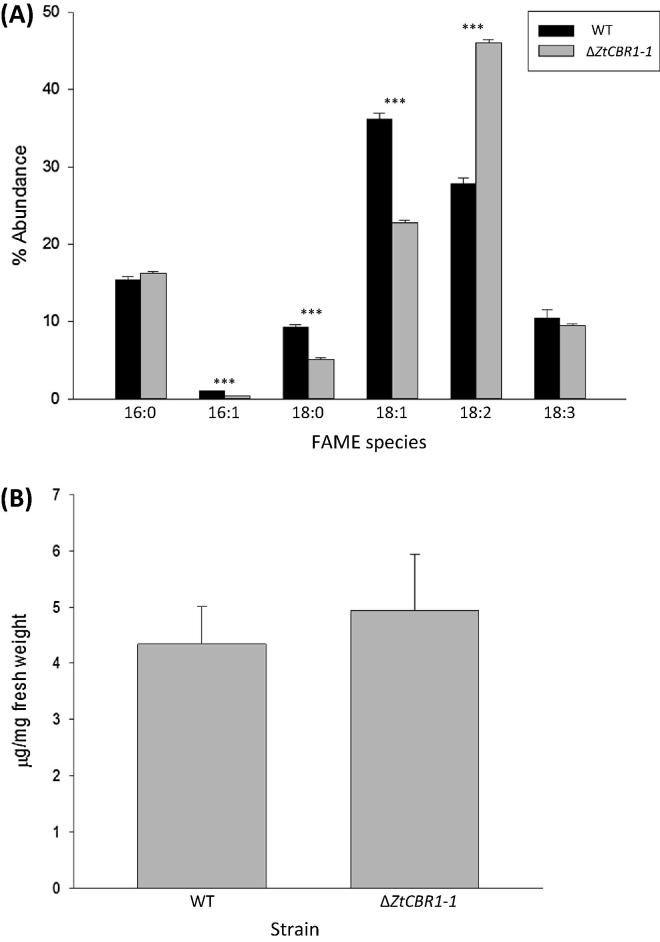
Δ*ZtCBR1* mutants have altered fatty acid methyl ester (FAME) profiles. (A) Mean relative abundance of the fatty acid methyl ester (FAME) species 16:0, 16:1, 18:0, 18:1, 18:2 and 18:3 in the WT and a Δ*ZtCBR1* strain. Bars represent standard error (^**^ *p* < 0.001). (B) Total FAME content of WT and the same Δ*ZtCBR1* strain expressed in ^***^ μg mg^−1^ of fresh weight. Bars represent standard error.

In order to assess whether *ZtCBR1* ablation had impacted on fatty acid biosynthesis by fungal cells, the fatty acid methyl ester (FAME) profile of the WT strain and Δ*ZtCBR1*-*1* were analysed using GC–MS. Δ*ZtCBR1*-*1* displayed a significant decrease in relative abundance of the fatty acid species 16:1, 18:0 and 18:1 (*p* < 0.001). This strain also displayed a significant increase in relative abundance of the polyunsaturated species 18:2 (*p* < 0.001). The relative abundances of the species 16:0 and 18:3 were not significantly changed relative to WT relative abundances (*p* > 0.05) (Fig. 6A). The total amount of FAMEs in Δ*ZtCBR1*-*1* was not significantly altered relative to WT levels (*p* > 0.05) (Fig. 6B).

### 3.6 Δ*ZtCBR1*-*1* displays an altered sphingolipid long chain base (LCB) profile

**Fig. 7:**
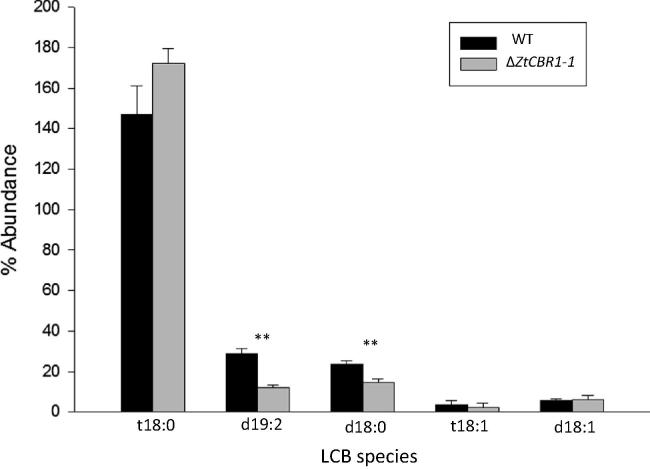
Δ*ZtCBR1* mutants have altered sphingolipid profiles. (A) Mean relative abundance of the sphingolipid long chain base (LCB) species trihydroxy 18:0 (t18:0), dihydroxy 19:2 (d19:2), dihydroxy 18:0 (d18:0), trihydroxy 18:1 (t18:1) and dihydroxy 18:1 (d18:1) in a WT and Δ*ZtCBR1* mutant strain. Bars represent standard error (^**^ *p* < 0.01).

In order to assess the effect of *ZtCBR1* ablation on sphingolipid biosynthesis, sphingolipid LCBs were analysed in the WT strain and Δ*ZtCBR1*-*1* using HPLC. The Δ*ZtCBR1*-*1* mutant exhibited a significant decrease in relative abundance of the LCB species dihydroxy 19:2 (d19:2) and dihydroxy 18:0 (d18:0) compared to the WT (*p* < 0.01). This strain also displayed an increase in the relative abundance of trihydroxy 18:0 (t18:0) that was approaching significance at the 5% level (*p* = 0.054) (Fig. 7).

### 3.7 Δ*ZtCBR1*-*1* displays an altered sterol profile

**Fig. 8:**
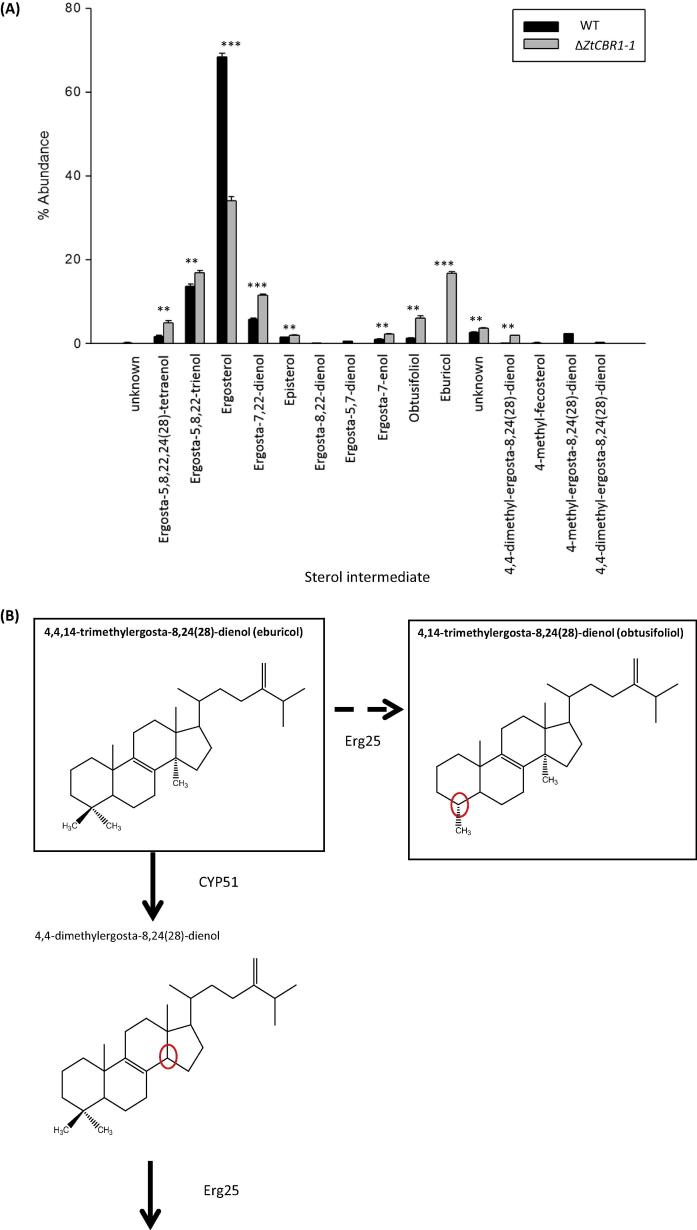
Δ*ZtCBR1* mutants have altered sterol profiles. (A) Mean relative abundance of all ergosterol and all intermediates in the ergosterol biosynthetic pathway identified for WT and a Δ*ZtCBR1* strain. Bars represent standard error, all deviations from WT levels in the Δ*ZtCBR1* strain were significant (^**^ *p* < 0.01, ^***^ *p* < 0.001). (B) Diagram depicting the reactions catalysed by the enzymes CYP51 and Erg25 during sterol biosynthesis. Solid arrow represents usual direction of biosynthetic pathway. Perforated arrow represents an alternative route for the CYP51 substrate, eburicol, which may be followed more frequently if CYP51 activity is compromised. Solid boxes surround compounds that accumulated in Δ*ZtCBR1* relative to the WT. Red circles mark the sites of enzymatic alterations at each sterol biosynthesis step. (For interpretation of the references to colour in this figure legend, the reader is referred to the web version of this article.)

In order to assess the effect of *ZtCBR1* ablation on sterol biosynthesis, the sterol profile of the WT and Δ*ZtCBR1*-*1* strains were analysed using GC–MS. Δ*ZtCBR1*-*1* displayed a significant reduction in the relative abundance of the final product of the sterol pathway, ergosterol (ergosta-5,7,22-trienol), relative to the WT (*p* < 0.001). Several intermediate compounds in the sterol biosynthesis pathway including ergosta-5,8,22,24(28)-tetraenol, ergosta-5,8,22-trienol, ergosta-7,22-dienol and obtusifoliol (14α-dimethyl-5α-ergosta-8,24(28)-dienol) were significantly increased in relative abundance in the Δ*ZtCBR1*-*1* strain compared to the WT (*p* < 0.01, *p* < 0.01, *p* < 0.001 and *p* < 0.01 respectively). The substrate of the enzyme CYP51 (eburicol (4,4,14-trimethylergosta-8,24(28)-dienol)), which is a target of azole antifungals, accumulated in Δ*ZtCBR1*-*1* but was not detected in the WT (*p* < 0.001) (Fig. 8).

## 4 Discussion

### 4.1 Unlike *S. cerevisiae* many filamentous fungal genomes have more than one microsomal *CBR* sequence

In several eukaryotes including plants and animals, the microsomal CBR-b_5_ electron transfer system has been shown to be important for the function of desaturase and hydroxylase enzymes involved in the biosynthesis of unsaturated fatty acids (UFAs), sterols and sphingolipids (Sperling et al., 1998; Uttaro, 2006; Poklepovich et al., 2012). Additionally, it is thought to be involved in certain CYP-catalysed reactions (Lamb et al., 1999, 2001). The genome of the model yeast *S. cerevisiae* contains only a single copy microsomal CBR sequence (Csukai et al., 1994; Truan et al., 1994). In the previously analysed filamentous fungus *M. alpina*, which is used in the industrial production of arachidonic acid, two microsomal CBRs have been identified in biochemical studies and structurally characterised (Sakuradani et al., 1999; Certik et al., 1999), though their functional importance to the organism is not known and the biochemical role of the secondary CBR is also unclear.

In the current study putative microsomal CBR sequences encoded in a range of fully sequenced fungal genomes were identified from a number of distantly related fungi including plant pathogens (biotrophs, hemibiotrophs and necrotrophs) and saprophytes. The mean number of putative microsomal CBR sequences identified was three, as was observed for *Z. tritici*, though some species contained considerably more. For instance *A. nidulans* contained seven and *Fusarium graminearum* contained six copies. However others, including *M. fijiensis* and *D. septosporum*, were more similar to *S. cerevisiae* having only a single predicted microsomal CBR sequence (Table 1). This is perhaps a little surprising given that these two fungi are also Dothideomycetes and are members of the genus *Mycosphaerellaceae* alongside *Z. tritici*. Intriguingly out of the five endophytic species analysed only the genome of one contained sequences similar to the *S. cerevisiae* CBRs. Though it is not possible to identify the precise reason for a larger number of microsomal CBR enzymes in certain fungal species without further functional studies, we could speculate that this larger number might be associated with a larger diversity of the terminal CBR electron acceptors. In particular the CYPs are known to be highly diversified amongst fungi, where they are thought to be important for metabolism of the diverse array of xenobiotics to which the organism may be exposed (Chen et al., 2014). It is interesting to note that the largest number of microsomal CBRs was found in the species *A. nidulans*, which is capable of colonising numerous environmental niches and therefore may require the ability to metabolise a more diverse array of xenobiotic compounds.

Our genome-wide analysis overall suggested no clear links between the numbers of predicted *CBR* genes with either particular pathogenic or saprophytic lifestyles. The exception to this is the five endophytic species analysed which frequently returned no significant sequence homologues (highest e value cut off used = e^−10^). Contrarily for one of these species, the rice endophyte *Harpophora oryzae*, the predominant difference between its genome and those of non-endophytic species that has been observed is relative expansion of numerous gene families, in particular those associated with transposable elements and carbohydrate metabolism. In fact, in this species only 10 gene families were found to be contracted relative to non-endophytes (though details of these families are not presented in the cited study) (Xu et al., 2014). Though only a speculation, it is possible that the selective pressures of an endophytic lifestyle may lead to a loss of genes associated with lipid metabolism like the CBRs. However, such an observation has yet to be formally tested.

### 4.2 *ZtCBR1* is constitutively expressed and required for full virulence on wheat leaves and asexual sporulation whereas *ZtCBR2* is not

In addition to the presence of canonical CBR domains in the retrieved sequences, PFam analysis also identified numerous putative microsomal CBRs with N-terminal b_5_ fusions. Though such fusions have been reported before (Yantsevich et al., 2008; Davis et al., 2002), little is known about their functions. *ZtCBR2* in *Z. tritici* was found to contain a b_5_ fusion domain. Intriguingly this gene shared a similar expression pattern to two neighbouring genes, including a putative *CYP*, annotated in the *Z. tritici* genome sequence as *CYP-24*, and a putative *hydroxyacyl coA dehydrogenase* (Fig. 2B). Given the presence of a b_5_ fusion domain and the observation that the CBR-b_5_ system may transfer electrons to CYPs, it is possible that this represents a discrete, co-regulated electron transfer chain.

Genes in this putative cluster were significantly up-regulated relative to their expression during growth in CDB in PDB and relative to either during growth in CDB or growth at all other infection time points on the fourth day of infection (*p* < 0.05) (Fig. 2B and C). Up-regulation of the genes in this cluster in the nutrient-rich medium PDB relative to CDB would suggest that this micro-region might be involved in metabolism of complex nutrient sources. However, low levels of expression of this cluster at later time points during plant infection when complex nutrient sources are released from necrotic host tissue would suggest that this is not the case.

An alternative hypothesis would be that this cluster is responsible for degradation of a compound common to numerous plant species, as PDB is derived from plant material. Degradation or metabolism of host-derived molecules by CYP enzymes has been shown to be important in various plant pathogenic fungi (Coleman et al., 2011; Pedrini et al., 2013; Miao et al., 1991). However, targeted deletion of the two genes *ZtCBR2* and *ZtCYP-24* did not lead to any reduction in virulence or asexual sporulation *in planta* (Supplementary Fig. S2) or any clearly evident phenotypic change *in vitro*. If ZtCBR2 is the primary electron donor for the enzymes in this cluster, it may be that the cluster is functionally redundant for plant infection. Furthermore, even if ZtCYP-24 is able to receive electrons from an alternate redox partner, this observation would indicate that alone it is not essential. Further characterisation of this cluster, including single and double deletions of both the *CYP* and the putative *hydroxyacyl coA dehydrogenase* would be needed to characterise its potential role in plant infection.

Despite the apparent high number and potential diversification of *CBR* sequences in filamentous fungi, in *Z. tritici* only one *CBR*, *ZtCBR1*, was highly expressed under both *in vitro* conditions and at all infection time points tested (Fig. 2A). This may indicate that it is the major *Z. tritici CBR* involved in processes described in other eukaryotes for members of this gene family. Further evidence from this comes from the observed biochemical and growth defects in Δ*ZtCBR1* strains and the delayed virulence and absence of asexual sporulation in infected wheat leaves (Figs. 4–8).

### 4.3 *ZtCBR1* is involved in fatty acid, sphingolipid and sterol metabolism in *Z. tritici*

In accordance with the various roles of the CBR-b_5_ electron transfer system in other eukaryotes, unsaturated fatty acid (UFA), sphingolipid and sterol profiles of Δ*ZtCBR1* strains were analysed and aberrations in all three of these pathways were observed (Figs. 6–8). Biosynthesis of UFAs proceeds via the insertion of double bonds between carbons of fatty acyl chains by desaturases, which are reliant on the CBR-b_5_ electron transfer system for reducing power. The first double bond is normally formed between the 9th and 10th carbons (the Δ9 position) of palmitic (16:0) or stearic (18:0) acid to make palmitoleic (16:1) or oleic (18:1) acid respectively. In all eukaryotes, this is carried out by a Δ9 desaturase (which is fused to b_5_ in fungi) that receives electrons from the CBR system (Uttaro, 2006; Tamura et al., 1976).This is the only desaturation event that takes place in *S. cerevisiae* as it only contains a single fatty acid desaturase, OLE1 (Stukey et al., 1990).

Many plants and fungi can carry out further desaturations, including *M. alpina* which contains Δ5, Δ6 and Δ12 desaturases, as well as a multifunctional desaturase and two additional Δ9 desaturases with differing substrate specificity (Knutzon et al., 1998; Sakuradani et al., 1999; Sakuradani and Shimizu, 2003; Kikukawa et al., 2013; MacKenzie et al., 2002; Wongwathanarat et al., 1999). In the current study, fatty acid methyl ester (FAME) derivatives of fatty acid species with either 16 or 18 carbons and up to 3 double bonds were investigated in a Δ*ZtCBR1* mutant strain, Δ*ZtCBR1*-*1*. It was found that the relative abundances of 18:1 and 16:1 were significantly depleted in this strain, suggesting that ZtCBR1 is an important redox partner for the *Z. tritici* Δ9 desaturase. However, the Δ9 desaturase substrates 18:0 and 16:0 did not significantly accumulate in Δ*ZtCBR1*-*1* relative to the WT. Conversely 18:0 decreased and 18:2, the product of Δ12 desaturation of 18:1, increased in relative abundance in this strain (Fig. 6A). It may be that the increase in 18:2 observed was a compensation for the perturbations in sterol and sphingolipid biosynthesis that were also observed in this strain. However, at this point and without protein enzyme activity studies, it is not possible to conclusively determine the precise fatty acid desaturase enzymes to which ZtCBR1 transfers electrons.

Sphingolipid biosynthesis also involves the activity of desaturase enzymes. Sphingolipids are composed of two distinct portions, a fatty acid and a long chain base (LCB), which are amide-linked via a variety of possible head groups. The LCB is an aliphatic amino alcohol which may vary in the number of double bonds or hydroxyl groups that it possesses. In addition to the desaturases involved in LCB biosynthesis, the hydroxylases that are involved also rely on the CBR-b_5_ system for electrons. In this study sphingolipid LCBs were analysed. Two distinct LCB desaturases are known to exist in fungi, the Δ4 and the Δ8 desaturase (Oura and Kajiwara, 2008; Beckmann et al., 2003). It is possible that ZtCBR1 is involved in electron transfer to these enzymes in *Z. tritici* as it was found that dihydroxy 19:2 (d19:2) was depleted in Δ*ZtCBR1*-*1* relative to the WT. Though there was no increase in the precursor of this compound, d18:1, a close to statistically significant increase in the relative abundance of trihydroxy 18:0 (t18:0) was observed (*p* = 0.054) (Fig. 7). This may be a compensation for the lack of d19:2 as an increase in hydroxylated LCBs may offset some of the effects of depletion of saturated LCBs. A reduction in the amount of dihydroxy 18:0 (d18:0) provides further evidence for this as it is the precursor of t18:0, which may be produced via additional hydroxylation. However, again it is not possible to precisely determine which sphingolipid desaturase or hydroxylase enzymes ZtCBR1 provides electrons to without further functional studies.

Sterol biosynthesis involves various desaturases and hydroxylases and the two cytochrome P450s (CYPs), CYP51 and CYP61 (Lepesheva and Waterman, 2007; Alcazar-Fuoli et al., 2006; Bjorkhem and Leitersdorf, 2000; Kelly et al., 1997). *S. cerevisiae* only has three CYPs, including the sterol biosynthetic CYPs and CYP56, a dityrosine hydroxylase involved in sporulation (Briza et al., 1994). Intriguingly CPR, the cytochrome P450 reductase, usually thought to be the primary redox partner for CYPs, has been shown to be dispensable in *S. cervisiae* (Sutter and Loper, 1989). This may be due to use of the CBR-b_5_ system as an alternative redox partner for CYPs as it has been demonstrated that it can fully support CYP51 activity in a reconstituted cell-free system (Lamb et al., 1999). Disruption of *ZtCBR1* had a major effect on sterol biosynthesis. In Δ*ZtCBR1*-*1* the final product ergosterol was significantly depleted in relative abundance compared to the WT. The two pathway intermediates that increased in relative abundance most prominently in this strain were eburicol, the substrate of CYP51, and obtusifoliol, the product of 4 α demethylation of eburicol by Erg25, which usually acts downstream of CYP51 (Bard et al., 1996) (Fig. 8). This may indicate that ZtCBR1 is not only an alternative redox partner for CYP51 in *Z. tritici* but that it is necessary for its function.

Δ*ZtCBR1* was also found to accumulate ergosta-5,8,22 trienol which has been shown to accumulate in some CYP61-inhibited fungal strains (Loto et al., 2012). This may indicate that ZtCBR1 is also important for the function of this enzyme in *Z. tritici*. Finally, another intermediate, ergosta-7,22-dienol, was also shown to accumulate in Δ*ZtCBR1*. This is likely a result of decreased activity of the sterol Δ5 desaturase enzyme, ERG3, which requires CBR for electron transfer (Poklepovich et al., 2012; Kawata et al., 1985; Arthington et al., 1991) (Fig. 8).

Little is understood about the direct involvement of CBR in electron transfer to the fungal sterol biosynthesis CYPs CYP51 and CYP61, though it has been shown that disruption of the intermediate electron transfer enzyme b_5_ leads to an accumulation of a similar array of sterol intermediates in the Ascomycete yeast *Candida albicans* (Rogers et al., 2004). Further indication of the importance of this system for sterol biosynthesis comes from the observation that in *Schizosaccharomyces pombe*, Sre1, which is known to regulate sterol biosynthetic enzymes, also regulates CBR and b_5_ (Todd et al., 2006), and in humans CYP51 catalysis has been shown to be enhanced by the presence of b_5_ (Lamb et al., 2001). However, b_5_ has been shown to enhance CYP catalysed reactions independently of CBR via allosteric interaction (Porter, 2002), leading to the question of potential functional redundancy between the two electron donors CPR and CBR for CYP catalysis during sterol biosynthesis. In the current study, CBR enzyme functional ablation was shown to strongly affect both CYP-catalysed and the Δ5 desaturase-catalysed steps of the sterol biosynthetic pathway, which ultimately led to a reduction in relative abundance of the final product ergosterol. To our knowledge this represents the first direct observation of the effects of CBR functional ablation on sterol biosynthesis in a natural live cell system.

### 4.4 Growth and virulence defects in Δ*ZtCBR1* strains may be attributable to one or more of the various lipid metabolism abnormalities

Though it may not be possible to pinpoint the precise biochemical basis of the morphological, growth and virulence defects observed in Δ*ZtCBR1*, we can speculate that alterations in sphingolipid and sterol content were a major contributory factor. Sterols and sphingolipids are known to group together in biological membranes to form specific regions that have been termed lipid rafts (for a review see Simons and Sampaio, 2011). Lipid raft regions are hypothesised to be important sites for the attachment of specific membrane proteins involved in various cellular processes. For example, in *S. cerevisiae* several different sphingolipid and sterol biosynthesis mutants have shown deficiencies in Golgi trafficking (Proszynski et al., 2005).

Another important process in *S. cerevisiae* that involves formation of lipid rafts is mating. This involves polarisation of the plasma membrane to form a ‘schmoo tip’, in which lipid raft domains have been observed (Bagnat and Simons, 2002). Echoing this process, the fungus *C. albicans* has been shown to utilise lipid raft domains to form hyphae. Evidence for this comes from the observation of these regions at hyphal tips and from the formation of aberrant hyphae in spores exposed to sterol or sphingolipid biosynthesis-disrupting compounds (Martin and Konopka, 2004).

The morphological defects observed in Δ*ZtCBR1* strains could be representative of an underlying defect in lipid raft formation. Filamentous growth in this strain, though WT in appearance, was substantially slowed (Fig. 5). Due to their reduced LCB and ergosterol content Δ*ZtCBR1* spores may have been less frequently able to produce lipid raft domains, leading to less frequent hyphal extension resulting in slower overall growth. This is consistent with observations of reductions in particular sphingolipid LCB species and ergosterol rather than total ablation.

Other defects brought on by aberrations in lipid raft formation may be more specific to a pathogenic lifestyle. For instance, in several mammalian-pathogenic fungi, lipid raft-embedded proteins have been shown to be essential for adherence to host cells (Humen et al., 2011; Mittal et al., 2008). In the plant pathogen *F. graminearum*, the importance of lipid rafts in infection was demonstrated through disruption of the ceramide synthase gene (*Bar1*), essential for sphingolipid biosynthesis. The *F. graminearum* Δ*Bar1* mutant strains were unable to produce perithecia though hyphal differentiation and leaf penetration were still observed. Intriguingly in the Δ*Bar1* mutant strains generated in this study, sporulation resulted in the formation of shorter, less uniform spores with fewer cells than the WT (Rittenour et al., 2011). A similar observation was made for the Δ*ZtCBR1* mutants presented in the current study, which also showed these morphological defects (Fig. 4).

The highly reduced amount of sporulation in Δ*ZtCBR1* is particularly interesting given that this strain was eventually able to produce an extensive hyphal network *in vitro* (Fig. 5) and induce full necrosis of leaf tissue (Fig. 3A). This suggests that the normal virulence mechanisms that may elicit host cell death are still functionally intact in the Δ*ZtCBR1* strains but occur later, possibly as a consequence of the reduced hyphal growth rate. There are various possible explanations for subsequent loss of asexual sporulation in diseased leaves, including the influence of lipid signalling on developmental processes such as growth and proliferation. For instance, it has been demonstrated that in *S. cerevisiae* sphingolipid LCBs interact with the protein kinase Pkh1, which controls numerous processes including cell wall integrity and growth (Liu et al., 2005). Furthermore, lipid rafts are also known to mediate localisation of H-Ras, a key element of the mitogen activated protein kinase (MAPK) signalling pathway, to the correct sites in the cellular membrane systems (Anderson, 2006). This is intriguing because in *Z. tritici* disruption of the MAPK-encoding gene *MgFus3* led to a lack of pycnidiation *in vitro* (Cousin et al., 2006). In light of data presented in the current study, it is possible that this MAPK is influenced by cellular lipid content. Future analysis should involve sequential disruption of *Z. tritici* genes involved in biosynthesis of specific lipids and lipid-derived signalling molecules.

### 4.5 Conclusion

By characterising members of the *CBR* family in *Z. tritici* this study has demonstrated for the first time the importance of these genes in regulating infection-related processes in a plant pathogenic fungus. To our knowledge, this is the first time that these genes have been functionally characterised in fungi other than *S. cerevisiae* and *M. alpina*. Defects in pathways thought to require enzymes that receive electrons from CBR-b_5_ observed in Δ*ZtCBR1* ultimately led to an almost complete lack of asexual sporulation *in planta*. Thus, processes dependent upon particular CBR-b_5_ electron transfers in *Z. tritici* may represent important new targets for future disease intervention.

## Figures and Tables

**Table 1:** Number and distribution of *Saccharomyces cerevisiae* CBR homologues in filamentous fungal genome sequences Below left: four plant endophytic fungi that had no sequences homologous to *S. cerevisiae* CBRs in their genomes. Fungal lifestyles were derived from Urban et al., 2015 (footnote a).

Sequence	Species	ScCBR1 aa identity (%)	ScCBR1 e value	TargetP mitochondrial prediction?	ScMCR1 aa identity (%)	ScMCR1 e value	b_5_ fusion domain?	Fungal lifestyle
XP_003854385.1 (*ZtCBR1*)	*Zymoseptoria tritici*	49	1.00E−82	N	–	–	N	Hemibiotrophic plant pathogen
XP_003852872.1 (*ZtCBR2*)	*Zymoseptoria tritici*	39	1.00E−63	N	–	–	Y	Hemibiotrophic plant pathogen
XP_003852910.1	*Zymoseptoria tritici*	39	3.00E−57	Y	47	2.00E−78	N	Hemibiotrophic plant pathogen
XP_003847738.1 (*ZtCBR3*)	*Zymoseptoria tritici*	33	1.00E−30	N	–	–	N	Hemibiotrophic plant pathogen
XP_756793.1	*Ustilago maydis*	46	6.00E−82	N	–	–	N	Biotrophic plant pathogen
XP_759922.1	*Ustilago maydis*	40	8.00E−41	Y	36	3.00E−62	N	Biotrophic plant pathogen
XP_001799967.1	*Stagonospora nodorum*	51	5.00E−95	N	–	–	N	Necrotrophic plant pathogen
XP_001806619.1	*Stagonospora nodorum*	46	2.00E−70	N	–	–	Y	Necrotrophic plant pathogen
XP_001801691.1	*Stagonospora nodorum*	39	3.00E−51	Y	46	5.00E−76	N	Necrotrophic plant pathogen
XP_003322373.1	*Puccinia graminis*	48	5.00E−84	N	–	–	N	Biotrophic plant pathogen
XP_003319934.2	*Puccinia graminis*	38	1.00E−47	Y	40	6.00E−62	N	Biotrophic plant pathogen
CCA68189.1	*Piriformospora indica*	49	6.00E−93	N	–	–	N	Endophyte
CCA67532.1	*Piriformospora indica*	32	1.00E−46	Y	46	2.00E−69	N	Endophyte
XP_009850995.1	*Neurospora tetrasperma*	50	1.00E−80	N	–	–	N	Saprophyte
XP_009854993.1	*Neurospora tetrasperma*	46	2.00E−72	N	–	–	Y	Saprophyte
EGZ77533.1	*Neurospora tetrasperma*	42	1.00E−53	Y	49	3.00E−84	N	Saprophyte
XP_009856344.1	*Neurospora tetrasperma*	41	2.00E−53	N	–	–	N	Saprophyte
XP_009849163.1	*Neurospora tetrasperma*	32	2.00E−32	N	–	–	N	Saprophyte
XP_956601.1	*Neurospora crassa*	50	2.00E−80	N	–	–	N	Saprophyte
XP_965191.1	*Neurospora crassa*	46	4.00E−72	N	–	–	Y	Saprophyte
XP_964971.1	*Neurospora crassa*	42	1.00E−53	Y	43	1.00E−83	N	Saprophyte
XP_961775.1	*Neurospora crassa*	33	6.00E−31	N	–	–	N	Saprophyte
XP_007925194.1	*Mycosphaerella fijiensis*	44	9.00E−80	N	–	–	N	Hemibiotrophic plant pathogen
XP_007926128.1	*Mycosphaerella fijiensis*	40	3.00E−55	Y	45	2.00E−73	N	Hemibiotrophic plant pathogen
XP_007922376.1	*Mycosphaerella fijiensis*	30	2.00E−29	Y	32	2.00E−50	N	Hemibiotrophic plant pathogen
XP_385028.1	*Fusarium graminearum*	46	1.00E−71	N	–	–	Y	Nectrotrophic plant pathogen
ESU10740.1	*Fusarium graminearum*	46	4.00E−59	N	–	–	Y	Nectrotrophic plant pathogen
XP_383723.1	*Fusarium graminearum*	43	9.00E−69	N	–	–	Y	Nectrotrophic plant pathogen
XP_382313.1	*Fusarium graminearum*	42	4.00E−65	N	–	–	Y	Nectrotrophic plant pathogen
XP_381102.1	*Fusarium graminearum*	39	2.00E−56	Y	46	2.00E−75	N	Nectrotrophic plant pathogen
XP_387123.1	*Fusarium graminearum*	32	1.00E−34	N	–	–	N	Nectrotrophic plant pathogen
XP_385079.1	*Fusarium graminearum*	30	4.00E−27	N	–	–	N	Nectrotrophic plant pathogen
EME45766.1	*Dothistroma septosporum*	44	5.00E−84	N	–	–	N	Hemibiotrophic plant pathogen
EME46111.1	*Dothistroma septosporum*	38	1.00E−48	Y	42	3.00E−69	N	Hemibiotrophic plant pathogen
EME38673.1	*Dothistroma septosporum*	33	5.00E−37	Y	31	4.00E−46	N	Hemibiotrophic plant pathogen
EFQ24978.1	*Colletotrichum graminicola*	50	1.00E−81	N	–	–	N	Hemibiotrophic plant pathogen
EFQ25098.1	*Colletotrichum graminicola*	46	8.00E−72	N	–	–	Y	Hemibiotrophic plant pathogen
EFQ27839.1	*Colletotrichum graminicola*	41	2.00E−57	Y	46	2.00E−76	N	Hemibiotrophic plant pathogen
EFQ36452.1	*Colletotrichum graminicola*	33	1.00E−33	Y	34	3.00E−53	N	Hemibiotrophic plant pathogen
XP_007700426.1	*Cochliobolus sativus*	54	5.00E−89	N	–	–	N	Nectrotrophic plant pathogen
XP_007700318.1	*Cochliobolus sativus*	44	2.00E−69	N	–	–	Y	Nectrotrophic plant pathogen
XP_007704621.1	*Cochliobolus sativus*	40	8.00E−53	Y	46	2.00E−76	N	Nectrotrophic plant pathogen
EMD92868.1	*Cochliobolus heterostrophus*	53	8.00E−89	N	–	–	N	Nectrotrophic plant pathogen
EMD88525.1	*Cochliobolus heterostrophus*	44	2.00E−69	N	–	–	Y	Nectrotrophic plant pathogen
EMD86858.1	*Cochliobolus heterostrophus*	40	7.00E−53	Y	46	2.00E−76	N	Nectrotrophic plant pathogen
EPQ65715.1	*Blumeria graminis*	45	3.00E−66	N	–	–	N	Biotrophic plant pathogen
CCU81251.1	*Blumeria graminis*	44	2.00E−64	N	–	–	N	Biotrophic plant pathogen
EPQ62420.1	*Blumeria graminis*	43	2.00E−58	N	–	–	N	Biotrophic plant pathogen
CCU78450.1	*Blumeria graminis*	42	3.00E−58	N	–	–	N	Biotrophic plant pathogen
XP_001208762.1	*Aspergillus terreus*	51	7.00E−88	N	–	–	N	Facultative parasite/saprophyte
XP_001218611.1	*Aspergillus terreus*	45	1.00E−74	N	–	–	Y	Facultative parasite/saprophyte
XP_001215899.1	*Aspergillus terreus*	44	1.00E−69	N	–	–	Y	Facultative parasite/saprophyte
Q0CRD8.2	*Aspergillus terreus*	41	3.00E−49	Y	49	2.00E−86	N	Facultative parasite/saprophyte
XP_001212924.1	*Aspergillus terreus*	39	5.00E−47	Y	48	7.00E−83	N	Facultative parasite/saprophyte
XP_001214268.1	*Aspergillus terreus*	31	8.00E−31	Y	39	1.00E−67	N	Facultative parasite/saprophyte
XP_663970.1	*Aspergillus nidulans*	51	6.00E−88	N	–	–	N	Facultative parasite/saprophyte
Q5AZB4.2	*Aspergillus nidulans*	51	7.00E−88	N	–	–	N	Facultative parasite/saprophyte
XP_661466.1	*Aspergillus nidulans*	47	3.00E−77	N	–	–	Y	Facultative parasite/saprophyte
CBF75218.1	*Aspergillus nidulans*	47	5.00E−77	N	–	–	Y	Facultative parasite/saprophyte
XP_658036.1	*Aspergillus nidulans*	43	4.00E−57	Y	48	1.00E−86	N	Facultative parasite/saprophyte
XP_682189.1	*Aspergillus nidulans*	38	5.00E−59	N	–	–	Y	Facultative parasite/saprophyte
CBF82301.1	*Aspergillus nidulans*	37	6.00E−38	N	–	–	Y	Facultative parasite/saprophyte
XP_663990.1	*Aspergillus nidulans*	32	9.00E−31	N	–	–	N	Facultative parasite/saprophyte
XP_755738.2	*Aspergillus fumigatus*	49	2.00E−86	N	–	–	N	Facultative parasite/saprophyte
XP_753636.1	*Aspergillus fumigatus*	45	4.00E−75	N	–	–	Y	Facultative parasite/saprophyte
XP_748717.1	*Aspergillus fumigatus*	44	1.00E−70	N	–	–	Y	Facultative parasite/saprophyte
XP_750202.1	*Aspergillus fumigatus*	43	4.00E−50	Y	47	5.00E−79	N	Facultative parasite/saprophyte

Endophytes with no sequences retrieved by SsCBR1								
*Epichloe festucae*								Microsomal
*Ascocoryne sarcoides*								Mitochondrial
*Penicillium aurantiogriseum*								
*Harpophora oryzae*								

a Fungal lifestyle source, Urban et al., 2015 Nucleic Acids Research. Names given to the *Z. tritici CBR* genes referred to throughout the rest of this study are given underneath GenBank accessions.
