# Supplementary material for: Analysis of cytochrome b5 reductase-mediated metabolism in the phytopathogenic fungus Zymoseptoria tritici reveals novel functionalities implicated in virulence
Source: Fungal Genet Biol. 2015 Sep;82:69–84. doi: 10.1016/j.fgb.2015.05.008 (PMC4557397; doi:10.1016/j.fgb.2015.05.008)
Supplement: Supplementary data 1 [file mmc1.pdf]

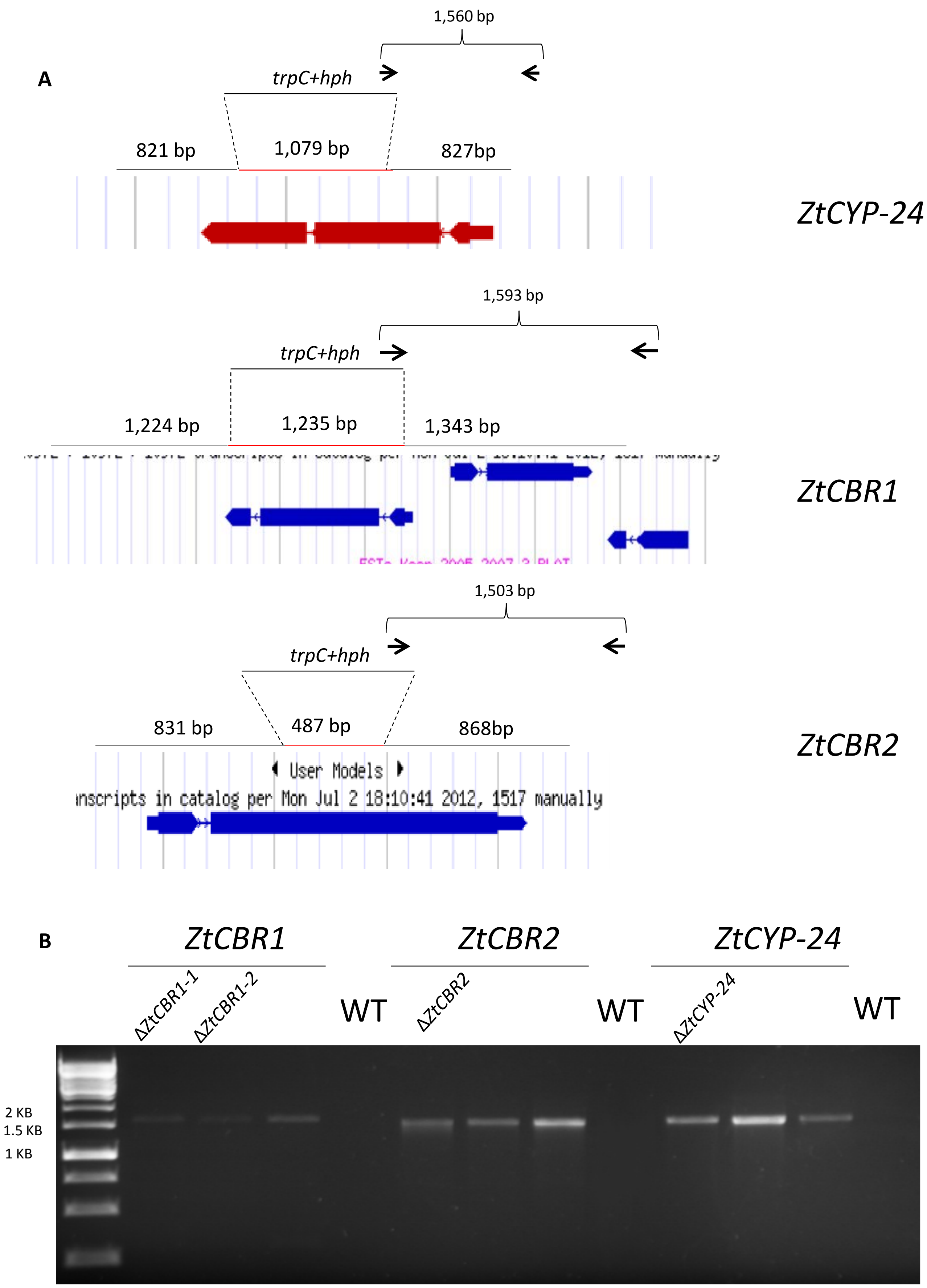

Supplementary Figure 1. Targeted gene disruption strategy and confirmation. (A) Positions of flanking sequences relative to the genes of interest in the *Z. tritici* genome, sizes of flanking sequences and sizes of regions to be replaced by the hygromycin resistance cassette under a *trpC* promoter (*trpC+hph*). Primers used for confirmation of successful gene disruption are marked with arrows and amplicon size is indicated above. (B) Confirmation of integration of *trpC+hph* at the correct location in the correct orientation using a primer from within *hph* and primer from within 200 bp of genomic DNA adjacent to one of the flanking sequences used to guide insertion. A single band of the correct amplicon size is indicative of successful gene targeting. WT gDNA was used as a control. Strains taken forward for further studies and their names are indicated above the relevant PCR lanes.

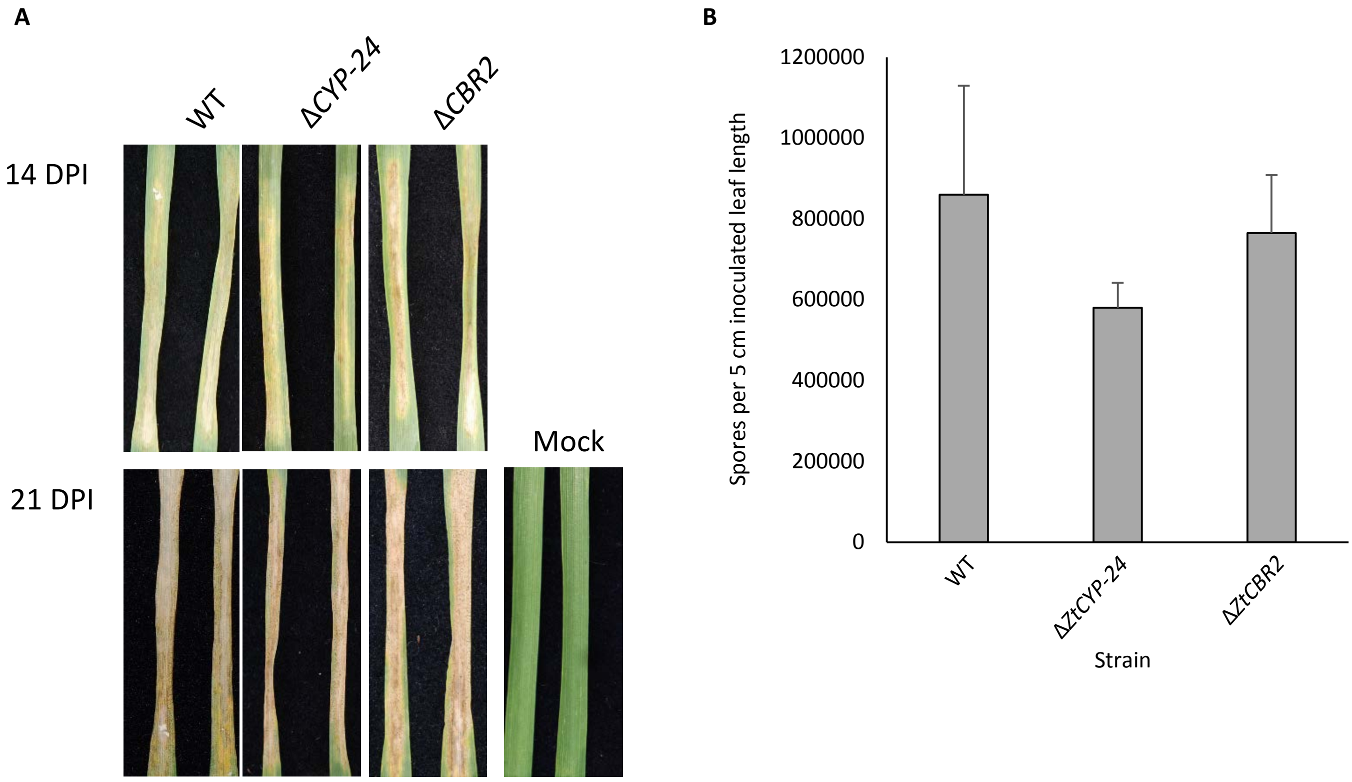

Supplementary Figure 2. Wheat leaf infection assays with Δ*ZtCYP-24* and Δ*ZtCBR2* strains

(A) Leaves infected with WT, Δ*ZtCYP-24* and Δ*ZtCBR2* after 14 and 21 DPI; mock-inoculated control leaves at 21 DPI are shown to the right. A total of 12 leaves per strain/mock were inoculated and two representative leaves are shown. (B) Mean number of spores (recovered by washing) per 5 cm length of inoculated leaf for WT, ΔZt*CYP-24* and Δ*ZtCBR2*.

Primer name	Sequence	Use	Restriction site
NB5.5-1F	aaaagatctTTGTCATTCGTGTAGCCTGTGT	Amplification of flanking sequence NB5.5-1 for targeting *Ztcbr1*	BglII
NB5.5-1R	aaaggcgcgccactagtACTTGCTGGAGAGACTCTGCG	Amplification of flanking sequence NB5.5-1 for targeting *Ztcbr1*	SpeI
NB5.3-1F	aaaggcgccATCTGTGGTCCTCCTCCTATGGTT	Amplification of flanking sequence NB5.3-1 for targeting *Ztcbr1*	NarI
NB5.3-1R	aaaggcgcgccCAGGCAAAAAAGGCGTCTATGA	Amplification of flanking sequence NB5.3-1 for targeting *Ztcbr1*	SgsI
HYG-N1F	aaaactagtGATATTGAAGGAGCATTTTTTGG	Amplification of hygromycin resistance cassette to clone into pNOV2114	SpeI
HYG-N1R	aaaggcgccCTACTACTATTCCTTTGCCCTCG	Amplification of hygromycin resistance cassette to clone into pNOV2114	NarI
CBR2 F1 F	aagagctcACGATGAACGCATCGGTTTCATCACGATGCTACGGTTTTG	Amplification of flanking sequence CBR2F1 for targeting *Ztcbr2*	SacI
CBR2 F1 R	aaggtaccAAGTGAATTGCAGCCCACGCA	Amplification of flanking sequence CBR2F1 for targeting *Ztcbr2*	KpnI
CBR2 F2 F	aaaagcttGCCGACATTCGCGGTCCCAA	Amplification of flanking sequence CBR2F2 for targeting *Ztcbr2*	HindIII
CBR2 F2 R	aaactagtCGAGTACCGCGCTTTGGGGG	Amplification of flanking sequence CBR2F2 for targeting *Ztcbr2*	SpeI
CYP-24 F1 F	aaaagcttATGTCGAGAGATCGTGAGTTGTTTGCA	Amplification of flanking sequence CYP-24F`1 for targeting *Ztcyp-24*	HindIII
CYP-24 F1 R	aatctagaGATTCAACTTTGCACTGCTAGAAGTGAAGAT	Amplification of flanking sequence CYP-24F`1 for targeting Ztcyp-24	XbaI
CYP-24 F2 F	aaggtaccGATGACTGCTGTTGATCTCCTTCGAA	Amplification of flanking sequence CYP-24F2 for targeting *Ztcyp-24*	KpnI
CYP-24 F2 R	aagagctcTTGCTTGAACACTTGGCTTTCGGT	Amplification of flanking sequence CYP-24F2 for targeting Ztcyp-24	SacI
CBR1 + Hyg F	GCTGATCTGACCAGTTGCCT	CBR1 KO confirmation	
CBR1 + Hyg R	CAATCGTGAGGGGTTCAGCT	CBR1 KO confirmation	
CBR2 + Hyg F	AGTCGTCCAGGCGGTGAGCA	CBR2 KO confirmation	
CBR2 + Hyg R	GGCGAAGCTCTCCGGCCATC	CBR2 KO confirmation	
CYP-24 + Hyg F	AGTCGTCCAGGCGGTGAGCA	CYP-24 KO confirmation	
CYP-24 + Hyg R	GGCCCTCCCTGAACTCCCCA	CYP-24 KO confirmation	

Supplementary Table 1 All primers used in this study with added adenines followed by restriction sites in lower case at the 5’ end.

Fungal genome	URL
Zymoseptoria tritici	http://genome.jgi-psf.org/Mycgr3/Mycgr3.home.html
Ustilago maydis	http://genome.jgi.doe.gov/Ustma1/Ustma1.home.html
Stagonospora nodorum	http://genome.jgi.doe.gov/Stano2/Stano2.home.html
Puccinia graminis	http://genome.jgi-psf.org/Pucgr1/Pucgr1.home.html
Piriformospora indica	http://genome.jgi-psf.org/Pirin1/Pirin1.home.html
Neurospora tetrasperma	http://genome.jgi-psf.org/Neute_matA2/Neute_matA2.home.html
Neurospora crassa	http://genome.jgi-psf.org/Neucr2/Neucr2.home.html
Mycosphaerella fijiensis	http://genome.jgi.doe.gov/Mycfi2/Mycfi2.home.html
Fusarium graminearum	http://genome.jgi.doe.gov/Fusgr1/Fusgr1.home.html
Dothistroma septosporum	http://genome.jgi-psf.org/Dotse1/Dotse1.home.html
Colletotrichum graminicola	http://genome.jgi-psf.org/Colgr1/Colgr1.home.html
Cochliobolus sativus	http://genome.jgi.doe.gov/Cocsa1/Cocsa1.home.html
Cochliobolus heterostrophus	http://genomeportal.jgi-psf.org/CocheC5_3/CocheC5_3.home.html
Blumeria graminis f. sp. *hordei*	http://genome.jgi.doe.gov/Blugr1/Blugr1.home.html
Aspergillus terreus	http://genome.jgi.doe.gov/Aspte1/Aspte1.home.html
Aspergillus nidulans	http://genome.jgi.doe.gov/Aspnid1/Aspnid1.home.html
Aspergillus fumigatus	http://genome.jgi.doe.gov/Aspfu1/Aspfu1.home.html

Supplementary Table 2 URLs of all genome sequences incorporated into genome-wide analysis of *CBR* genes.
